# Hierarchical classification-based pan-cancer methylation analysis to classify primary cancer

**DOI:** 10.1186/s12859-023-05529-0

**Published:** 2023-12-08

**Authors:** Youpeng Yang, Qiuhong Zeng, Gaotong Liu, Shiyao Zheng, Tianyang Luo, Yibin Guo, Jia Tang, Yi Huang

**Affiliations:** 1 Medicine School, Sun Yat-sen University, Shenzhen, 518107 China; 2 Geneplus-Shenzhen Institute, Shenzhen, 518118 China; 3 NHC Key Laboratory of Male Reproduction and Genetics, Guangdong Provincial Reproductive Science Institute (Guangdong Provincial Fertility Hospital), Guangzhou, 510062 China; 4 School of Medicine, Jinan University, Guangzhou, 510632 China

**Keywords:** Cancer, Classification, Cluster analysis, Machine learning

## Abstract

Hierarchical classification offers a more specific categorization of data and breaks down large classification problems into subproblems, providing improved prediction accuracy and predictive power for undefined categories, while also mitigating the impact of poor-quality data. Despite these advantages, its application in predicting primary cancer is rare. To leverage the similarity of cancers and the specificity of methylation patterns among them, we developed the Cancer Hierarchy Classification Tool (CHCT) using the idea of hierarchical classification, with methylation data from 30 cancer types and 8239 methylome samples downloaded from publicly available databases (The Cancer Genome Atlas (TCGA) and the Gene Expression Omnibus (GEO)). We used unsupervised clustering to divide the classification subproblems and screened differentially methylated sites using Analysis of variance (ANOVA) test, Tukey-kramer test, and Boruta algorithms to construct models for each classifier module. After validation, CHCT accurately classified 1568 out of 1660 cases in the test set, with an average accuracy of 94.46%. We further curated an independent validation cohort of 677 cancer samples from GEO and assigned a diagnosis using CHCT, which showed high diagnostic potential with generally high accuracies (an average accuracy of 91.40%). Moreover, CHCT demonstrates predictive capability for additional cancer types beyond its original classifier scope as demonstrated in the medulloblastoma and pituitary tumor datasets. In summary, CHCT can hierarchically classify primary cancer by methylation profile, by splitting a large-scale classification of 30 cancer types into ten smaller classification problems. These results indicate that cancer hierarchical classification has the potential to be an accurate and robust cancer classification method.

**Supplementary Information:**

The online version contains supplementary material available at 10.1186/s12859-023-05529-0.

## Introduction

The aggressive and unpredictable spread pattern of malignant tumors always poses a considerable challenge to patients and medical personnel. Traditional cancer detection techniques are usually invasive, single-target, and difficult to detect cancer at early stages. However, with the development of genetic testing technology, molecular diagnostic technologies are playing an increasingly important role in cancer detection and early diagnosis. As a method for molecular tumor diagnosis, DNA tumor detection is divided into genetic mutation detection and methylation detection. Nevertheless, normal human cells undergo continual mutations, some of which involve cancer-related genes. However, only a limited number of these mutations actually contribute to tumor development. This situation can result in false positives and may suggest the challenge of distinguishing the tissue origin of tumors from various organs solely based on genetic mutations [1]. In contrast, tumor development and progression are accompanied by changes in DNA methylation patterns, which are generally tissue-specific. Thus, there are extensive differences in DNA methylation patterns between normal and tumor cells from different tissues and organs.

DNA methylation is a fundamental epigenetic mark that governs cell identity and gene expression changing occurs in early carcinogenesis [2]. Tumor suppressor genes are inactivated by hypermethylation [3]. By the contrary, hypomethylation can cause genomic instability and facilitates transformation into tumor cells [4]. These altered methylation distributions lead to the suppression of tumor oncogene expression and an increase in proto-oncogene expression, further promoting tumorigenesis and development. DNA methylation patterns are consistent with the cells or tissues where they originate, implying that detection of tumor-specific DNA methylation may serve as a feasible approach for developing a cancer detection test [5]. Many studies have shown that the DNA methylation patterns are tumor type-specific [6] and tissue-specific [7] and are changed in the early stage of cancer development across the whole genome [8]. Therefore, the DNA methylation patterns could be a valuable marker for cancer detection and determination of tissue origin of tumors.

In this regard, various studies have focused on identifying tumor based on DNA methylation patterns. Koelsche et al. constructed a random forest model to classify soft tissue and bone tumors using a dataset of 1077 methylation profiles. Their findings demonstrated the potential of DNA methylation-based sarcoma classification for research and future diagnostic applications [9]. Hao et al. evaluated the utility of DNA methylation for differentiating tumor tissue from normal tissue in four common cancers. They found that they could differentiate cancerous tissue from normal tissue with 95% accuracy [10]. Moran et al. established a random forest classifier of cancer type based on the microarray DNA methylation signatures in a training set of 2790 tumor samples of known origin representing 38 tumor types and including 85 metastases showing high diagnostic potential [6]. Capper et al. present a comprehensive approach for the DNA methylation-based classification of central nervous system tumors across all entities and age groups. Predictions from that classifier changed 12% of the original neuropathology diagnoses in an independent validation cohort [11]. Shimizu et al. established the Cancer Cell-of-Origin methylation panel using the methylation data of the 28 types of cancer in TCGA (The Cancer Genome Atlas), which showed high sensitivity and specificity [12]. Modhukur et al. used 24 cancer types and 9303 methylome samples to construct machine learning classifiers to discriminate metastatic, primary, and non-cancerous methylome samples with an average accuracy of 99% [13].

The classification method used in these studies, as mentioned above, is flat classification, which requires all features that are informative enough to distinguish all involved cancer types to be found at once. However, (i) in flat classification, the increase in the number of categories to be recognized may lead to a decrease in classification accuracy, especially in the case of relatively homogeneous category classifications. (ii) There is also no way for flat classification to provide predictive power in the face of cancer types not included in the classifier, even if such cancer is somewhat associated with certain cancer in the classifier. In fact, the natural hierarchy of the data could have high classification values. Many important real-world classification problems are naturally reduced to hierarchical classification problems in which the classes to be predicted are organized into a hierarchy of classes-typically lung cancer, squamous cell lung carcinoma and lung adenocarcinoma, ignoring those parent–child class relationships could lose some vital information. (iii) In flat classification, poor data quality in some categories may affect all other categories, which in turn affects the performance of the overall models [14].

In addition, methylation data, as a complex dataset, can reflect the similarities and origins of different tumors. It can be used to organize cancer entities into a tree structure and hierarchically classify them based on their similarities, thereby achieving a more complex classification.

In this study, instead of flattening out and ignoring those inner hierarchies, we considered the similarity of the cancers and developed a Cancer Hierarchy Classification Tool (CHCT) using the idea of hierarchical classification that splits a large-scale classification problem of 30 cancer types into a set of small classification problems to classify primary cancer by the methylation profile hierarchically. To carry out our study, we utilized 8239 tissue samples spanning 30 common primary cancers from the open-access database TCGA and GEO (Gene Expression Omnibus), split into training set and test set with a 4:1 ratio. UPGMA (unweighted pair group method with arithmetic mean), an unsupervised algorithm for grouping data based on overall similarity, was used to determine the architecture of CHCT, which divided 30 cancer types into 12 groups. Thus, the large problem of classifying 30 types of cancer was divided into ten subproblems. The Random Forest algorithm has excellent robustness, outstanding performance in handling high-dimensional data, overfitting prevention capabilities, and effectiveness in handling imbalanced data. Thus, we used the Random Forest algorithm to develop our models with the training set samples to solve these ten subproblems. Each classification problem corresponds to a classification model. We assess the model’s performance on a held-out test cohort. Finally, we evaluated our models by testing them on an independent test cohort consisting of multiple tumor types from the GEO database.

**Fig. 1:**
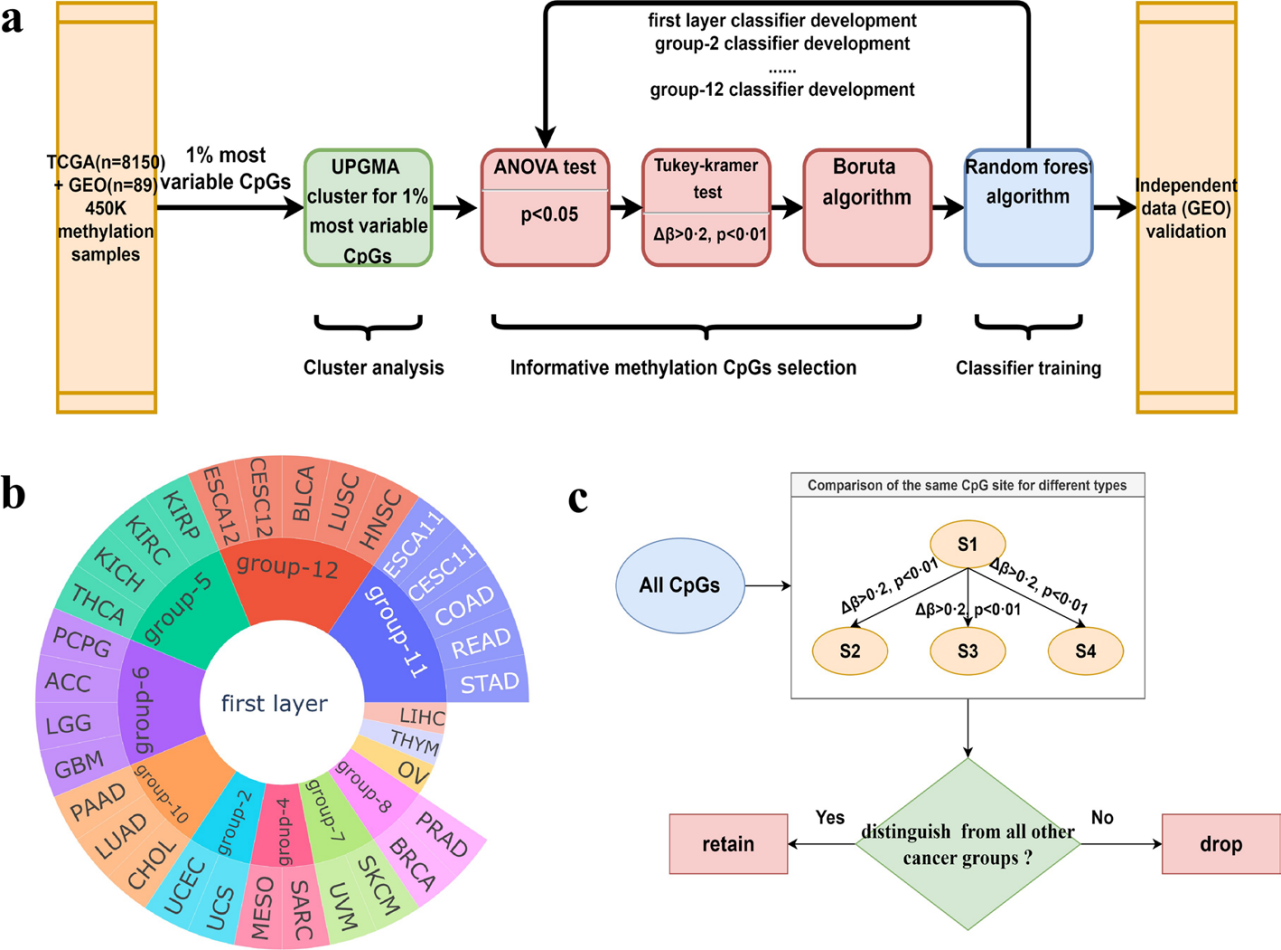
Schematic of the study method, architecture of CHCT, and identification of the informative CpG sites by Tukey-kramer test. **a** Raw methylation cancer data across 30 cancer types were downloaded from TCGA and GEO. After data preprocessing, the data was clustered by UPGMA to divide cancer groups. The first layer classifier labels were established. Then, we firstly used ANOVA test to select the probes with a significant difference. We further used the Tukey-kramer test to screen probes with differences from all other 11 groups. Boruta algorithm was applied to select the most informative CpGs. Finally, we built a predictive model containing these CpGs as features, and the model was tested using test set. The second layer classifier was built similarly. To assess the models’ adaptability, we collected the independent primary cancer methylation data cohort from GEO to test models. **b** The architecture diagram of CHCT. CHCT can be viewed as a tool with a two-tier architecture. The data to be predicted is first predicted by the first layer of CHCT, and the result of this layer is used to further mobilize the next layer of the prediction model to get the final prediction results (For OV, THYM, and LIHC, the first layer prediction model gives the prediction directly). **c** The one vs all other approach seeks to screen the CpG sites that distinguish each cancer type from all other cancer types. In this illustration, consider a hypothetical differential consisting of four cancer types. The pairwise differential approach aims to identify the best markers for differentiating each possible cancer pair

## Methods

### Overview

CHCT can be viewed as a tool with a two-tier architecture. The data to be predicted is first predicted by the first layer of CHCT, and the result of this layer is used to further mobilize the next layer of the prediction model to get the final prediction results (For OV (Ovarian Cancer), THYM (Thymoma), and LIHC (Liver Cancer), the first layer prediction model gives the prediction directly). The overall procedure of CHCT comprises five main steps: (i) sample acquisition from TCGA and GEO, and data preprocessing; (ii) clustering samples using the UPGMA algorithm; (iii) screening biomarkers using ANOVA (Analysis of variance) test, Tukey-Kramer test, and Boruta algorithm; (iv) training random forest models to classify cancer types and constructing CHCT; and (v) validating CHCT. An overview of the study workflow is illustrated in Fig. 1.

### Sample acquisition from TCGA and GEO, and data preprocessing

In this study, raw primary cancer methylation data from the Illumina Human Methylation 450 BeadChip (450K) methylation platforms were obtained from TCGA and GEO. The UCSC Xena browser [15] was used to download methylation data based. Since only a few OV (n = 10) were profiled using 450K array in TCGA, we supplemented with OV samples (n = 89) from GEO (GSE65820, GSE81224). We collected a total of 8239 samples (TCGA = 8150 and GEO = 89) from 30 types of cancer, including ACC (Adrenocortical Cancer), BLCA (Bladder Cancer), BRCA (Breast Cancer), CESC (Cervical Cancer), CHOL (Bile Duct Cancer), COAD (Colon Cancer), ESCA (Esophageal Cancer), GBM (Glioblastoma), HNSC (Head and Neck Cancer), KICH (Kidney Chromophobe), KIRC (Kidney Clear Cell Carcinoma), KIRP (Kidney Papillary Cell Carcinoma), LGG (Lower Grade Glioma), LIHC, LUAD (Lung Adenocarcinoma), LUSC (Lung Squamous Cell Carcinoma), MESO (Mesothelioma), OV, PAAD (Pancreatic Cancer), PCPG (Pheochromocytoma and Paraganglioma), PRAD (Prostate Cancer), READ (Rectal Cancer), SARC (Sarcoma), SKCM (Melanoma), STAD (Stomach Cancer), THCA (Thyroid Cancer), THYM, UCEC (Endometrioid Cancer), UCS (Uterine Carcinosarcoma), UVM (Uveal melanomas). The beta value was used to estimate methylation levels derived from methylated and unmethylated probe intensities using the formula M/(M + U + 100), where M and U are fully methylated and fully unmethylated intensities, respectively. Since the CpG sites in different datasets varied, only probes shared across all included datasets for each analysis were used for classifier training and testing. Moreover, we excluded probes on sex chromosomes and probes with missing values from methylation data. To assess the model’s adaptability, we collected the independent data cohort consisting of the Illumina Human Methylation 450 BeadChip primary tumor methylation data from GEO.

### Clustering samples using the UPGMA algorithm

We took several steps to preprocess our data effectively. First, we excluded subtypes that either had fewer than five samples or contributed less than 10% to the total number of samples for a given cancer type. Additionally, we excluded subtypes with ambiguous information. To reduce the dimensionality of our data and make it more amenable to result visualization without significant information loss, we calculated the mean methylation fraction for each subtype within each cancer type, resulting in clustered data. Subsequently, we divided the raw data into 103 distinct groups based on clinical subtypes. To identify the most informative CpG sites, we selected the top 1% of CpG sites that exhibited the highest variability across all samples. Next, we used the UPGMA [16] clustering algorithm, based on the Pearson correlation coefficient, to cluster the 103 groups. This clustering approach allowed us to visually represent the relationships among these groups. To determine the optimal number of clusters, we employed the silhouette coefficient [17] as a reference. The silhouette coefficient helped us assess the quality of clustering and make informed decisions about the number of clusters, ensuring the reliability of our results. The fanning diagram was plotted using ggtree [18]. All data preprocessing was conducted in Python version 3.10 and clustering was performed in R version 4.1.2.

### Screening biomarkers using ANOVA test, Tukey-Kramer test, and Boruta algorithm

Methylation data of 12 groups (a total of 8239 samples collected from 30 types of cancer) were analyzed to screen the group-specific probes for the first layer. We firstly applied ANOVA test to find any statistical differences in probes between the means of the 12 groups of cancer types, probes with p-value less than 0.5 were screened. Then, the Tukey-kramer test was performed for pairwise comparison of 12 groups aiming to screen probes with differences from the all the other 11 groups using the cut-off of the Tukey-corrected *p*-value adjusted for multiple comparisons < 0.01 and the absolute mean methylation difference between the compared groups of > 0.2 (${\Delta \beta }$ > 0.2, $p<$0·01). In some cases, such as group-10, we gradually decreased ${\Delta \beta }$ to 0.15 or 0.1 to ensure sufficient CpG sites were selected. Similarly, we identified biomarkers of groups in the second layer that contained at least two cancer types (group-2, group-4, group-5, group-6, group-7, group-8, group-10, group-11, and group-12). Noting the markers of ESCA11, STAD, COAD, READ, ESCA12, HNSC and LUSC were screened by gradually decreasing ${\Delta \beta }$ to 0.15 or 0.1 (Additional file 1: Table S2). All data processing was performed in Python version 3.10. To further reduce the feature’s dimension, Boruta algorithm was applied [19]. Boruta is an improved feature selection method based on the features importance of random forest classifier which is the estimator of Boruta. Boruta algorithm will generate random shaded features to be added to the original data, and compare the importance of the shaded features and the original features, then iterate several times, and finally compare the number of times the original features outperform the shaded features according to the binomial distribution to decide whether to keep or eliminate the features, so that the final features obtained can play a positive role in the prediction of the model. Boruta_py v 0.3, a python implementation of Boruta feature selection, was used. We used the same approach to execute Boruta Feature Selection for group-2, group-4, group-5, group-6, group-7, group-8, group-10, group-11 and group-12.

### Training random forest models to classify cancer types and constructing CHCT

We first randomly split each type of cancer samples into training set that was participated in model training and test set that was not involved in model construction throughout with a 4:1 ratio. Then, we reduced the dimension of these train data (ANOVA test, Tukey-kramer’s test and Boruta algorithm were used) to ensure that the model is trained with a modest number of features. The Random Forest model [20], where classifiers comprise many weak classifiers, was used to develop the cancer classifier incorporating all 6579 train samples of the 12 groups. The classification is performed by merging the decisions of all weak classifiers and then predicting the tumor types (by most common prediction from all weak classifiers). Since the number of methylome samples from each class and type of cancer was imbalanced, we set the parameter class weight = ’not-balanced’ to address unequal class size. After learning curve parameters tuning, the Random Forest generates 200 binary decision trees (classification and regression trees, CART) as weak classifiers. On the held-out 1660 test dataset, which the model did not see during training, the random forest model achieved an overall accuracy of 98.13%. To evaluate the robustness of the predicted model, the 5-fold cross-validation was performed on the model, showing a cross-validation score of 0.972. In 5-fold cross-validation, the data set is first randomly divided into five-folds, and then the i-th (i = 1, 2, 3, 4, 5) fold is used as the validation set to verify the random forest model constructed with the remaining (5–1) folds as the training set. This process is repeated k times until each fold is set as the validation set. The receiver operating characteristic curve shows a high AUC (0.999) in predicting 12 groups of 30 cancer types [21]. This result indicates a high separating capacity. We used the same approach to develop classifiers for group-2, group-4, group-5, group-6, group-7, group-8, group-10, group-11 and group-12. All data processing and model training were performed in Python version 3.10.

### Validating CHCT

To access the robustness of the classifier algorithm, we use the external independent validation cohort obtained from the GEO database. The model was evaluated using the top-k differential diagnosis accuracy, which measures how accurately the ground truth label is found in the k highest confidence predictions of the model. This allows us to potentially use the top predictions of CHCT for a given sample to narrow down the origin of the tumor to a handful of possible types. CHCT defaults to top-2² accuracy as the output accuracy: CHCT would select the top-2 of the first layer classifier firstly. If the top-2 of the first layer contains multi-classification groups such as group-5, group-6, group10, group11, or group12, CHCT would further select the top-2 of these groups in the second layer. Overall, the result of CHCT can be anywhere from 2 to 4. To visualize heterogeneity between samples, we used Uniform Manifold Approximation and Projection (UMAP) [22], which is a dimension reduction technique.

## Result

### The hierarchical structure of the CHCT recapitulates lineage relationships among cancer types

After completing data collection and processing, we first clustered the methylation data consisting of 30 cancer types from TCGA and GEO. The raw data was divided into 103 groups based on the clinical subtypes. We further used an unsupervised agglomerative algorithm (UPGMA) to cluster the 103 subtypes of 30 kinds of cancers by the top 1% most variable CpG site among them. These cancer classes were assigned to 12 categories relating to their Pearson similarity.

**Fig. 2:**
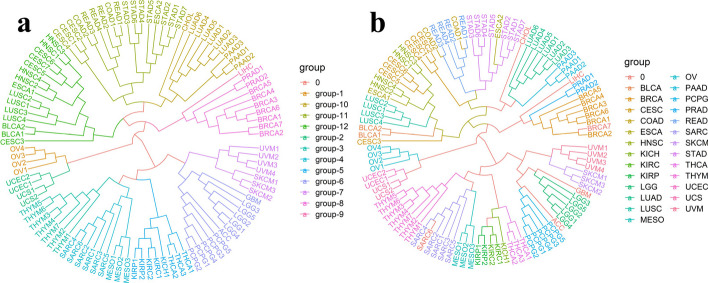
The result of clustering. **a** The result fanning diagram of unsupervised agglomerative clustering (UPGMA), reflecting that the cancers were clustered together in a certain lineage or tissue relationship. Cancer types are indicated by edge colors. **b** Considering the result of the cluster and silhouette coefficient of each cancer, we grouped 30 cancer types into 12 groups. Cancer groups are indicated by edge colors

For each cancer, its subtypes cluster in close proximity to each other, reflecting the homogeneity of different subtypes of cancer. Since ESCA adenocarcinomas and CESC adenocarcinomas are distinct from ESCA squamous cell carcinomas and CESC squamous cell carcinomas respectively, we separated the ESCA dataset and CESC dataset into four subtypes: ESCA adenocarcinoma (ESCA11), ESCA squamous carcinoma (ESCA12), CESC adenocarcinoma (CESC11) and CESC squamous carcinoma (CESC12). The result of the cluster is shown in Fig. 2. Strikingly, the clustering results showed that cancers were grouped together in a certain lineage or tissue relationship, and also showed a clear distinction between squamous carcinoma and adenocarcinoma [7, 23]. OV, UCEC and UCS cluster closely. But the relationship between OV, UCEC, and UCS is not as close as that of UCEC and UCS according to the silhouette coefficient. MESO and SARC cluster together, where mesothelioma can be considered as a type of sarcoma [24]. UVM and SKCM agglomerate in a union, as both cancer types derive from melanocytes that share the same embryonic origin and display the same cellular function [25]. Kidney-related cancers such as KICH, KIRP and KIRC, and THCA are clustered together, indicating interactions between kidney cancer and thyroid cancer [26]. ACC, LGG, GBM, and PCPG are lumped together, which are relatively homogeneous diseases [27]. PRAD and BRCA are very close. Some research has shown that there may be a link between prostate cancer and breast cancer due to certain common genetic mutations [28–30]. A family history of breast cancer is associated with an increased risk of prostate cancer. Certain common genetic mutations may contribute to these cancers. PAAD, LUAD, and CHOL cluster together as adenocarcinomas, as do STAD, ESCA11, READ, COAD, and CESC11. Squamous carcinomas such as CESC12, ESCA12, BLCA, LUSC and HNSC agglomerate together. Finally, considering the result of the cluster and silhouette coefficient of each cancer, we grouped 30 cancer types into 12 groups. Table 1 shows the group of these cancers. Overall, UPGMA showed generally well-separated clusters according to our analysis.

**Table 1:** Components of each group

Group	Components
Group-1	OV
Group-2	UCS, UCSC
Group-3	THYM
Group-4	MESO, SARC
Group-5	KIRP, KIRC, KICH, THCA
Group-6	PCPG, ACC, LGG, GBM
Group-7	UVM, SKCM
Group-8	BRCA, PRAD
Group-9	LIHC
Group-10	PAAD, LUAD, CHOL
Group-11	STAD, READ, COAD, $\text {CESC}^*$, $\text {ESCA}^*$
Group-12	HNSC, LUSC, BLCA, CESC$$^{**}$$, ESCA$$^{**}$$

$$^{*}$$Adenocarcinoma

$$^{**}$$Squamous carcinoma

### The methylation CpGs selected demonstrate cancer type specificity

**Fig. 3:**
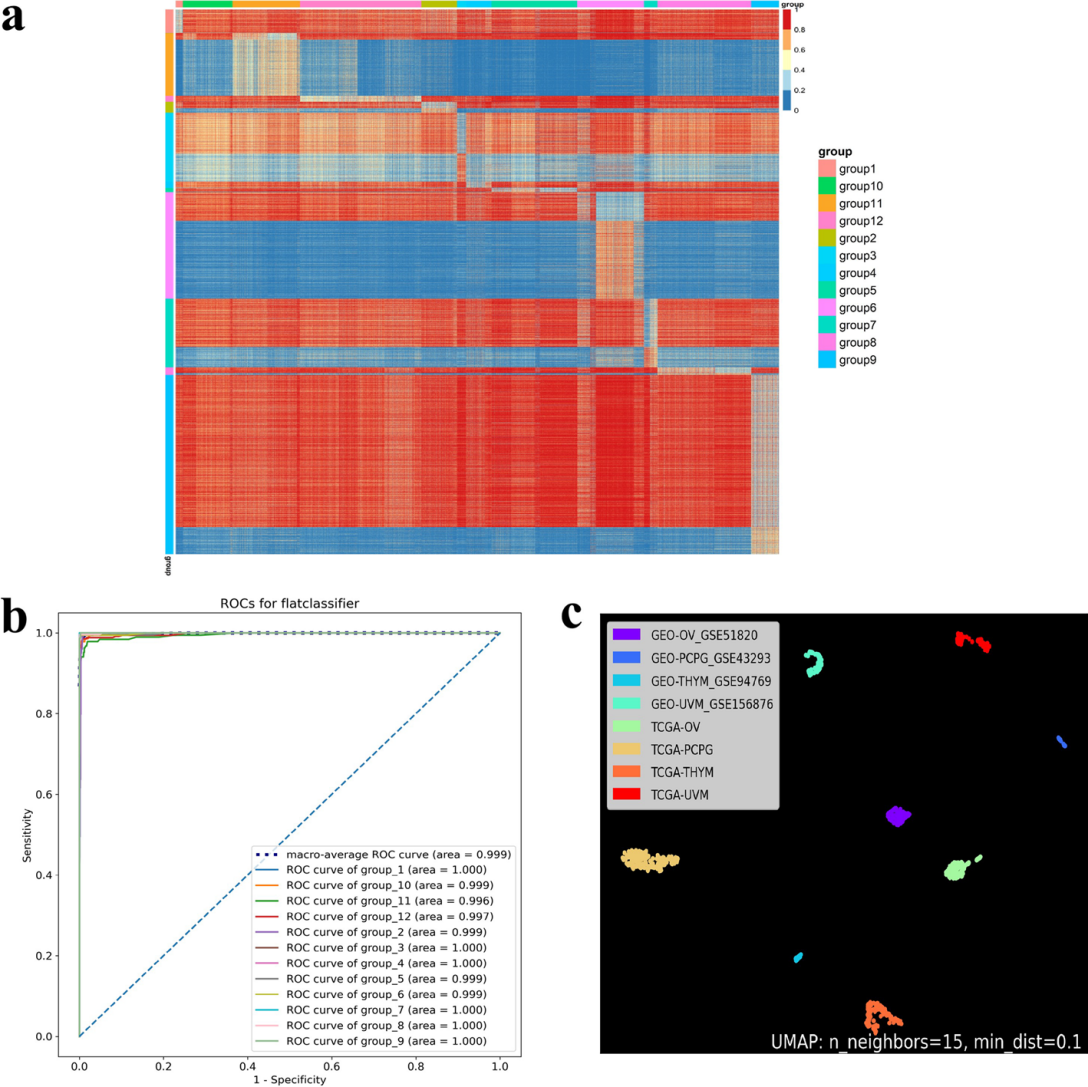
CHCT can identify the cancers well using the screen-out methylation signature. **a** Heat map depicts the methylation level of retained probes of first layer classifier, reflecting clear distinction among the 12 cancer groups. Upper annotation indicates sample types of 12 cancer groups. Left-sided annotation indicates 12 cancer-specific methylation panels. **b** ROC curve shows the high AUC in predicting 12 cancer types using the methylation signature. **c** UMAP plot shows a high degree of heterogeneity between the same type samples from TCGA and GEO

We conducted a differential methylation analysis on the 12 classes defined above to identify the most informative CpG sites for cancer-type classification. To achieve this, we used the ANOVA test, Tukey-Kramer test, and Boruta algorithm. Firstly, we used the ANOVA test to select probes with a significant difference. We then applied the Tukey-Kramer test to retain CpGs that could best distinguish one cancer group from all other cancer groups. From the 12 groups, we identified 13,103 probes that were statistically significant. We then used the Boruta algorithm to screen for more informative CpG sites, which resulted in the retention of 8061 probes. The heatmap shown in Fig. 3 displayed the differences between the 12 groups in each probe, and the selected methylated CpGs demonstrated significant tumor type specificity.

We further selected the features of the group-2, group-4, group-5, group-6, group-7, group-8, group-11, and group-12 of the second layer using the same procedure, resulting in the identification of 284, 238, 986, 1078, 339, 456, 874, and 1481 probes, respectively. The above selected markers are sufficiently discriminatory for each cancer type (Additional file 1: Fig. S1).

### CHCT can clearly classify the types of cancer

We initially split each type of cancer sample randomly into a training set, which was used to construct the model, and a test set, which was not involved in the model building, at a ratio of 4:1. The first layer of the CHCT classification model was constructed using the random forest algorithm, and the entire training data set with 8061 features was used to train the constructed model to classify 12 cancer groups consisting of 30 cancer types. The resulting model was then tested using the test dataset, and it achieved a 5-fold cross-validation score of 0.972 and an area under the receiver operating characteristic curve of 0.999, indicating a satisfactory predictive power. Of the 1660 cases, 1629 were correctly classified, resulting in an average accuracy of 98.13%. The precision and recall for the majority of cancer types were higher than 0.97. We used the same approach to construct the random forest models for the second layer, and their performance is shown in Additional file 1: Tables S1–S12 and Figs. S1–S2. Our results showed that these classifiers could perform well, with some achieving a maximum area under the curve (AUC) for the group-2, group-4, group-7, group-8, and group-10 classifiers (AUC = 1.000) and a minimum AUC for the group-11 classifier (AUC = 0.971). The highest 5-fold cross-validation score was achieved by the group-8 classifier (cv-score = 0.999), while the lowest score was achieved by the group-11 classifier (cv-score = 0.877). CHCT was able to accurately predict 1568 out of 1660 samples from the test set, achieving an accuracy rate of 94.5%. Our findings demonstrate that CHCT has a high diagnostic potential with high accuracy.

### CHCT is also universal to other methylation platform data

To evaluate the adaptability of our model, we collected independent cancer methylation data (n = 505) from the GEO cohort. We used top-$\textrm{k}^{2}$ to assess the performance of CHCT, which defaults to top-$2^{2}$ accuracy as the output accuracy. (Details of the evaluation method can be found in the Methods section.) Our trained model performed well without tuning or domain adaptation. Specifically, 13 cancer types achieved over 90% prediction accuracy, and the average accuracy of 19 cancer types reached 91.48%. Table 2 shows the specific prediction results.

We performed UMAP clustering on the same cancer data from different databases. Data from TCGA were mainly used for model construction and testing, and data from GEO were mainly used to verify model generalizability. We found a high degree of heterogeneity between the same type samples from TCGA and GEO. This suggests that different processing methods may have been used between different databases. However, our tool performs relatively well in different data processing methods. In brief, our models are capable of generalizing to diverse data sources and processing methods not encountered during model training or probe normalization.

**Table 2:** Independent validation cohort

Cancer type	GSE	Sample size	Auccary (%)
ACC	GSE77804	2	100
PAAD	GSE74071	14	100
HNSC	GSE67114	8	100
LUSC	GSE121849	20	100
KICH	GSE156932	8	75
KIRC	GSE156932	2	100
LIHC	GSE113019	18	100
LUAD	GSE66836	164	100
SKCM	GSE140169	46	97.83
UVM	GSE156876	5	100
MESO	GSE175769	10	90
OV	GSE51820	85	74.12
PCPG	GSE43293	22	100
PRAD	GSE157272	23	100
SARC	GSE89041	15	66
THYM	GSE94769	11	72
THCA	GSE77804	11	81.82
BRCA	GSE52865	40	97.50

### CHCT can provide some predictive power for cancer types not covered by the classifier

We utilized CHCT to predict outcomes for two representative datasets: the medulloblastoma dataset (GSE75153) and the pituitary tumor dataset (GSE147548). As expected, 100% of the predictions for medulloblastoma were concentrated in the group-6 category, in spite of medulloblastoma being a clinical subtype of glioma not covered by the TCGA dataset GBM and LGG. In addition, the classification of pituitary tumors in the CHCT dataset was also all clustered in group-6. Since pituitary tumors, like ACC, are endocrine adenomas composed of neuronal cells, it is logical for them to cluster in group-6. Hierarchical classifiers have the potential to offer predictive power for new tumors that were previously unknown.

## Discussion

Considering the similarities between tumors, we used the strategy of hierarchical classification that splits a complete multi-class problem into a set of smaller classification problems to build CHCT and achieved robust predictive performance.

In contrast to CHCT, we built a flat classification model using the same scenario, resulting in 3612 markers after screening. A random forest model was then built and achieved an accuracy of 93.23%, with a 5-fold cross-validation score of 0.932. And we also compare the CHCT with the 2 classifier that are used for primary cancer classification [31, 32]. In Wei’s research, They train a deep learning classifier to predict cancer type based on patterns of somatic passenger mutations detected in whole genome sequencing of 2606 tumours representing 24 common cancer types produced by the PCAWG Consortium and achieve an accuracy of 91% on held-out tumor samples. In Binsheng’s research, they developed a neural network framework using the expression of a 150-gene panel to infer the tumor tissue-of-origin for 15 common solid tumor cancer types and achieves an average prediction sensitivity and precision of 93.36% and 94.07%, respectively. The results demonstrated that, under circumstances where it leverages its unique advantages, the hierarchical classification model achieve comparable or even superior performance to these flat classification model by breaking down the problem into smaller units, classes that are not taxonomically related to a given class of interest are not considered in the classification process, leading to more focused and accurate results.

Apart from its performance advantages, hierarchical classifiers also offer some predictive power for new or unknown tumors. While it may not be possible to classify these new tumors, the classifiers can still predict the upper classes, which can be clinically relevant in determining the appropriate treatment. For instance, similar androgen reduction treatments, such as testosterone antagonists, can be used to treat both prostate and breast cancer. The CHCT provides predictive results that suggest specific cancer foci or associated features, enabling individualized treatment recommendations.

The hierarchical classification can better adapt to dynamic changes. Later on, as the number of cancer types increases, the classification structure can be dynamically adjusted according to the similarity between the newly added cancers and the original ones.

Furthermore, the advantages of hierarchical classification are further magnified in cases where data quality in some categories may not be comparable to other categories, as poor data quality in a category in a flat classification model may affect all other categories. In contrast, hierarchical taxonomy ensures that taxonomically distinct classes are not susceptible to this phenomenon.

Although the multi-level classification model may lead to error propagation when the upper-level classifier produces an error result, errors are most likely confined to its class or near classes. Hence, the k highest confidence predictions designed to narrow down potential cancer types could be used as the output of CHCT.

There are still some samples in the independent validation cohort that cannot be correctly classified by the current version of CHCT. There are several reasons: (i) the train data used to build models does not cover the entire spectrum of all subtypes, and an increase in the number of subtypes could help identify more methylation subclasses. (ii) Batch effects are also an essential factor affecting the validation results. Batch effects are sub-groups of measurements that have qualitatively different behavior across conditions and are unrelated to the biological or scientific variables in a study. Different reagent dosages, chips, experiment instruments, operation personnel, etc., result in batch effect [33]. (iii) Due to partial probe missing in the TCGA data, we did not take into account the local methylation correlations (i.e., significant correlations in methylation levels in neighboring regions) in this study, as it could lead to the loss of information related to methylation cascade regions. We plan to address this issue using more sophisticated methods in our future research [34–36]. (iv) Moreover, according to UMAP cluster results, it can be determined that there are relatively obvious differences between samples from different databases, which can be attributed to the different methods used to correct and normalize methylation data [37]. Despite these challenges, CHCT has demonstrated better adaptability than other methods. For example, Daniel Xia et al. achieved an accuracy of 94.5% on 2575 samples from 28 cancer types in TCGA using only 53 CpG probes [38]. However, their model was focused on extracting the most informative and minimalist set of CpG biomarkers, and hence only applicable to TCGA cases. Nevertheless, CHCT still achieves good classification performance even on non-TCGA data.

In summary, we have developed the CHCT, a powerful tool for cancer classification that serves as proof-of-concept for hierarchical classification models in methylation analysis, advancing the application of hierarchical classification in translational medicine. We propose that hierarchical classification has the potential to be an accurate and robust cancer classification method with advantages that flat classification does not possess. Moreover, different feature selection algorithm can be used to build models in future. It makes sense that different algorithms and features for different subproblems can provide better results. Further research may be necessary to optimize model performance.

### Supplementary Information

**Additional file 1:** Contains heatmaps of methylation site markers, ROC curve plots of models, a table of sample information, and classification performance reports of models.

**Additional file 2:** Provides the ID of TCGA Samples.

## Data Availability

Publicly available datasets were analyzed in this study. This data can be found here: https://xenabrowser.net/datapages/. Sample code of 8150 sample we selected in the TCGA database were showed in Additional file 2 and the code of the sample from GEO are included in this article. Other data generated during the current study are available in its Additional file 1 and the Github repository: https://github.com/yyp1999/Cancer-Hierarchical-Classification-Tool.
