# Supplementary material for: Hierarchical classification-based pan-cancer methylation analysis to classify primary cancer
Source: BMC Bioinformatics. 2023 Dec 8;24:465. doi: 10.1186/s12859-023-05529-0 (PMC10709847; doi:10.1186/s12859-023-05529-0)
Supplement: Supplementary file 1 — Additional file 1: Contains heatmaps of methylation site markers, ROC curve plots of models, a table of sample information, and classification performance reports of models. [file 12859_2023_5529_MOESM1_ESM.doc]

**Supplementary figure 1:** The above nine graphs from left to right from top to bottom are heatmaps of methylation site markers for group-2, group-3, group-4, group-5, group-6, group-7, group-8, group-10, group-11, and group-12, respectively.


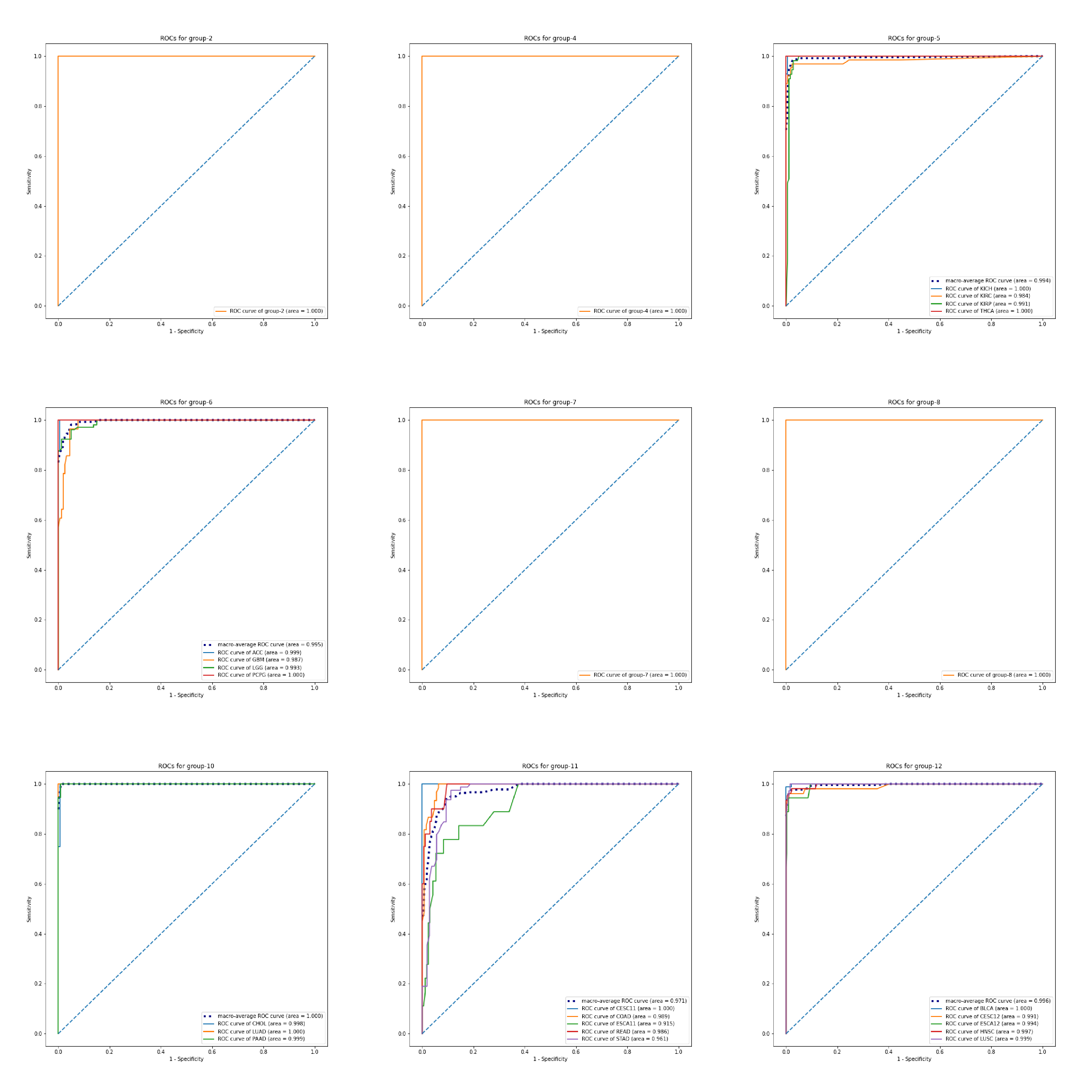


**Supplementary figure 2:** The above nine graphs from left to right from top to bottom are the ROC curves of classifiers for group-2, group-3, group-4, group-5, group-6, group-7, group-8, group-10, group-11, and group-12 respectively

**Supplementary table 1**: The full name of each cancer abbreviation and its sample size

| Abbreviation | Full name | Size |
| --- | --- | --- |
| ACC | Adrenocortical Cancer | 80 |
| BLCA | Bladder Cancer | 412 |
| BRCA | Breast Cancer | 782 |
| CESC | Cervical Cancer | 302 |
| CHOL | Bile Duct Cancer | 36 |
| COAD | Colon Cancer | 296 |
| ESCA | Esophageal Cancer | 177 |
| GBM | Glioblastoma | 140 |
| HNSC | Head and Neck Cancer | 528 |
| KICH | Kidney Chromophobe | 66 |
| KIRC | Kidney Clear Cell Carcinoma | 319 |
| KIRP | Kidney Papillary Cell Carcinoma | 275 |
| LGG | Lower Grade Glioma | 516 |
| LIHC | Liver Cancer | 377 |
| LUAD | Lung Adenocarcinoma | 458 |
| LUSC | Lung Squamous Cell Carcinoma | 370 |
| MESO | Mesothelioma | 87 |
| OV | Ovarian Cancer | 99 |
| PAAD | Pancreatic Cancer | 184 |
| PCPG | Pheochromocytoma and Paraganglioma | 179 |
| PRAD | Prostate Cancer | 498 |
| READ | Rectal Cancer | 98 |
| SARC | Sarcoma | 261 |
| SKCM | Melanoma | 105 |
| STAD | Stomach Cancer | 395 |
| THCA | Thyroid Cancer | 507 |
| THYM | Thymoma | 124 |
| UCEC | Endometrioid Cancer | 431 |
| UCS | Uterine Carcinosarcoma | 57 |
| UVM | Uveal melanomas | 80 |

**Supplementary table 2:** The markers of ESCA11, STAD, COAD, READ, ESCA12, HNSC and LUSC were screened by gradually decreasing ∆β to 0.15 or 0.1

|  | ∆β>0.1 | ∆β>0.15 | ∆β>0.2 |
| --- | --- | --- | --- |
| CESC11 | 47152 | 24258 | 10595 |
| ESCA11 | 2067 | 298 | 48 |
| STAD | 2430 | 106 | 4 |
| COAD | 881 | 70 | 4 |
| READ | 3069 | 455 | 54 |
| CESC12 | 13073 | 4297 | 1306 |
| ESCA12 | 317 | 10 | 0 |
| HNSC | 876 | 57 | 1 |
| LUSC | 2285 | 311 | 36 |
| BLCA | 21615 | 7737 | 2617 |

**Supplementary table 3:** Classification report of the first layer classifier

|  | precision | recall | f1-score | support |
| --- | --- | --- | --- | --- |
| group_1 | 1 | 0.916666667 | 0.956521739 | 24 |
| group_2 | 0.972222222 | 0.979020979 | 0.975609756 | 143 |
| group_3 | 0.994845361 | 0.960199005 | 0.97721519 | 201 |
| group_4 | 0.963855422 | 0.996884735 | 0.980091884 | 321 |
| group_5 | 0.956989247 | 0.978021978 | 0.967391304 | 91 |
| group_6 | 1 | 1 | 1 | 23 |
| group_7 | 0.966666667 | 0.983050847 | 0.974789916 | 59 |
| group_8 | 0.995614035 | 0.991266376 | 0.993435449 | 229 |
| group_9 | 0.994680851 | 1 | 0.997333333 | 187 |
| group_10 | 1 | 0.978723404 | 0.989247312 | 47 |
| group_11 | 1 | 0.992277992 | 0.996124031 | 259 |
| group_12 | 1 | 0.953125 | 0.976 | 64 |
| accuracy |  |  | 0.984830097 | 1648 |
| macro avg | 0.987072817 | 0.977436415 | 0.981979993 | 1648 |
| weighted avg | 0.985139304 | 0.984830097 | 0.984827959 | 1648 |

**Supplementary table 4:** Classification report of the group_2 classifier

|  | precision | recall | f1-score | support |
| --- | --- | --- | --- | --- |
| UCEC | 0.988636364 | 1 | 0.994285714 | 87 |
| UCS | 1 | 0.916666667 | 0.956521739 | 12 |
| accuracy |  |  | 0.98989899 | 99 |
| macro avg | 0.994318182 | 0.958333333 | 0.975403727 | 99 |
| weighted avg | 0.990013774 | 0.98989899 | 0.989708263 | 99 |

**Supplementary table 5:** Classification report of the group_4 classifier

|  | precision | recall | f1-score | support |
| --- | --- | --- | --- | --- |
| MESO | 1 | 1 | 1 | 18 |
| SARC | 1 | 1 | 1 | 53 |
| accuracy |  |  | 1 | 71 |
| macro avg | 1 | 1 | 1 | 71 |
| weighted avg | 1 | 1 | 1 | 71 |

**Supplementary table 6:** Classification report of the group_5 classifier

|  | precision | recall | f1-score | support |
| --- | --- | --- | --- | --- |
| KICH | 0.875 | 1 | 0.933333333 | 14 |
| KIRC | 0.951612903 | 0.921875 | 0.936507937 | 64 |
| KIRP | 0.927272727 | 0.927272727 | 0.927272727 | 55 |
| THCA | 1 | 1 | 1 | 102 |
| accuracy |  |  | 0.961702128 | 235 |
| macro avg | 0.938471408 | 0.962286932 | 0.949278499 | 235 |
| weighted avg | 0.962354152 | 0.961702128 | 0.961715637 | 235 |

**Supplementary table 7:** Classification report of the group_6 classifier

|  | precision | recall | f1-score | support |
| --- | --- | --- | --- | --- |
| ACC | 0.9375 | 0.9375 | 0.9375 | 16 |
| GBM | 0.827586207 | 0.857142857 | 0.842105263 | 28 |
| LGG | 0.951923077 | 0.951923077 | 0.951923077 | 104 |
| PCPG | 1 | 0.972222222 | 0.985915493 | 36 |
| accuracy |  |  | 0.940217391 | 184 |
| macro avg | 0.929252321 | 0.929697039 | 0.929360958 | 184 |
| weighted avg | 0.941154423 | 0.940217391 | 0.94060818 | 184 |

**Supplementary table 8:** Classification report of the group_7 classifier

|  | precision | recall | f1-score | support |
| --- | --- | --- | --- | --- |
| UVM | 1 | 0.952380952 | 0.975609756 | 21 |
| SKCM | 0.941176471 | 1 | 0.96969697 | 16 |
| accuracy |  |  | 0.972972973 | 37 |
| macro avg | 0.970588235 | 0.976190476 | 0.972653363 | 37 |
| weighted avg | 0.974562798 | 0.972972973 | 0.973052875 | 37 |

**Supplementary table 9:** Classification report of the group_8 classifier

|  | precision | recall | f1-score | support |
| --- | --- | --- | --- | --- |
| BRCA | 1 | 1 | 1 | 157 |
| PRAD | 1 | 1 | 1 | 100 |
| accuracy |  |  | 1 | 257 |
| macro avg | 1 | 1 | 1 | 257 |
| weighted avg | 1 | 1 | 1 | 257 |

**Supplementary table 10:** Classification report of the group_10 classifier

|  | precision | recall | f1-score | support |
| --- | --- | --- | --- | --- |
| CHOL | 1 | 0.75 | 0.857142857 | 8 |
| LUAD | 0.989247312 | 1 | 0.994594595 | 92 |
| PAAD | 0.973684211 | 1 | 0.986666667 | 37 |
| accuracy |  |  | 0.98540146 | 137 |
| macro avg | 0.987643841 | 0.916666667 | 0.946134706 | 137 |
| weighted avg | 0.985672033 | 0.98540146 | 0.984427097 | 137 |

**Supplementary table 11:** Classification report of the group_11 classifier

|  | precision | recall | f1-score | support |
| --- | --- | --- | --- | --- |
| STAD | 1 | 0.888888889 | 0.941176471 | 9 |
| CESC11 | 0.869565217 | 1 | 0.930232558 | 60 |
| ESCA11 | 0.625 | 0.277777778 | 0.384615385 | 18 |
| READ | 1 | 0.55 | 0.709677419 | 20 |
| COAD | 0.844444444 | 0.962025316 | 0.899408284 | 79 |
| accuracy |  |  | 0.860215054 | 186 |
| macro avg | 0.867801932 | 0.735738397 | 0.773022023 | 186 |
| weighted avg | 0.855564646 | 0.860215054 | 0.841152804 | 186 |

**Supplementary table 12:** Classification report of the group_12 classifier

|  | precision | recall | f1-score | support |
| --- | --- | --- | --- | --- |
| BLCA | 1 | 0.975903614 | 0.987804878 | 83 |
| CESC12 | 1 | 0.903846154 | 0.949494949 | 52 |
| ESCA12 | 1 | 0.555555556 | 0.714285714 | 18 |
| HNSC | 0.920353982 | 0.981132075 | 0.949771689 | 106 |
| LUSC | 0.902439024 | 1 | 0.948717949 | 74 |
| accuracy |  |  | 0.948948949 | 333 |
| macro avg | 0.964558601 | 0.88328748 | 0.910015036 | 333 |
| weighted avg | 0.952966997 | 0.948948949 | 0.946245082 | 333 |
